# Fabrication of BP2T functionalized graphene *via* non-covalent π–π stacking interactions for enhanced ammonia detection†

**DOI:** 10.1039/d1ra06879b

**Published:** 2021-11-08

**Authors:** Hu Li, Tianbo Duan, Omer Sher, Yuanyuan Han, Raffaello Papadakis, Anton Grigoriev, Rajeev Ahuja, Klaus Leifer

**Affiliations:** Shandong Technology Centre of Nanodevices and Integration, School of Microelectronics, Shandong University 250101 Jinan China Hu.Li@sdu.edu.cn; Department of Materials Science and Engineering-Ångström, Uppsala University 75121 Uppsala Sweden Klaus.Leifer@angstrom.uu.se; TdB Labs 75121 Uppsala Sweden; Department of Physics and Astronomy-Ångström, Uppsala University 75120 Uppsala Sweden

## Abstract

Graphene has stimulated great enthusiasm in a variety of fields, while its chemically inert surface still remains challenging for functionalization towards various applications. Herein, we report an approach to fabricate non-covalently functionalized graphene by employing π–π stacking interactions, which has potentialities for enhanced ammonia detection. 5,5′-Di(4-biphenylyl)-2,2′-bithiophene (BP2T) molecules are used in our work for the non-covalent functionalization through strong π–π interactions of aromatic structures with graphene, and systematic investigations by employing various spectroscopic and microscopic characterization methods confirm the successful non-covalent attachment of the BP2T on the top of graphene. From our gas sensing experiments, the BP2T functionalized graphene is promising for ammonia sensing with a 3-fold higher sensitivity comparing to that of the pristine graphene, which is mainly attributed to the enhanced binding energy between the ammonia and BP2T molecules derived by employing the Langmuir isotherm model. This work provides essential evidence of the π–π stacking interactions between graphene and aromatic molecules, and the reported approach also has the potential to be widely employed in a variety of graphene functionalizations for chemical detection.

## Abstract 2

Non-covalent functionalization of graphene has been achieved by employing π–π stacking interactions, and it is promising for ammonia detection with greatly enhanced sensitivity.

## Graphical abstract

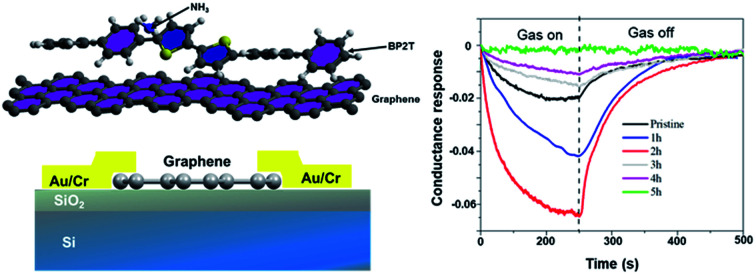

## Introduction

Graphene with its single-atom-thick two-dimensional (2D) conjugated structure has been extensively explored as an ideal material for chemical detection owing to its exceptional properties, *e.g.* superior electrical properties, ultra-large specific area, high mechanical sturdiness and good chemical stability.^1–6^ The high conductivity ensures graphene exhibits very little signal disturbance when working as a sensor, while the high chemical stability and strong mechanical performances ensure a long lifetime of such sensors.^7^ The intrinsic 2D structure gives graphene large specific surface areas that ensure a high sensitivity down to the level of even single molecule detection.^8^ All of these superior properties hold great promise for graphene to perform as a gas absorption material for sensors. Despite the great potentialities, the inert surface of the graphene results in numerous challenges for selective detection of various gases.^9–11^ To address this concern, various functionalization approaches have been exploited to conjugate selective ligands on its surface, including both covalent and non-covalent modifications.^12^

Non-covalent functionalization approach based on π–π stacking interactions shows unique advantages since it does not necessitate chemical modifications and thus can maintain the unique physical and structural properties of graphene.^13^ Aromatic molecules have been demonstrated useful for non-covalent functionalization by π–π stacking interactions.^14,15^ Owing to the aromatic structure, the molecules can strongly absorb onto the graphene surface, while at the same time, the functional groups of the aromatic molecules can be exposed and employed to recognize the target molecules directly.^16^ Therefore, the aromatic molecule based non-covalent functionalization of graphene is promising to be widely employed to achieve the surface modification of graphene towards chemical detections.

Ammonia (NH_3_) is a typical hazardous and irritant gas which can induces toxicity to the environment and sensitive ecosystems, therefore, a range of techniques have been employed to achieve the sensitive detection of NH_3_, including nanowires,^17,18^ metal oxide,^19^ reduced graphene oxide,^20^ laser-coupled spectroscopy,^21^*etc.* Despite the great potentialities as an ideal candidate for solid state gas sensors, graphene based ammonia sensors are still struggling as the sensor response is relatively much lower (typically less than 5%) as compared to other gas species sensed with pristine graphene. Even if the fact that many methods such as doping,^22^ defect insertion^23,24^ and chemical functionalization,^25^ have been applied, it is still a challenge to improve the sensitivity of graphene-based gas sensors to NH_3_.

In this work, we reported a systematical investigation of the graphene surface functionalized by employing π–π non-covalent stacking mechanism and also demonstrated its potential application in NH_3_ detection. Here, 5,5′-di(4-biphenylyl)-2,2′-bithiophene (BP2T) molecules are used for the non-covalent functionalization *via* strong π–π interactions between the aromatic structures of BP2T and graphene. Systematic analysis by utilizing different spectroscopic and microscopic characterizations was performed and the result confirms the successful non-covalent functionalization of the BP2T molecules on graphene surface. Moreover, our gas sensing experiments show that the BP2T molecule functionalized graphene demonstrates a 3-fold higher sensitivity for NH_3_ comparing to that of the pristine graphene, which opens up possibilities for graphene functionalization towards various chemical detections.

## Results and discussions

**Fig. 1:**
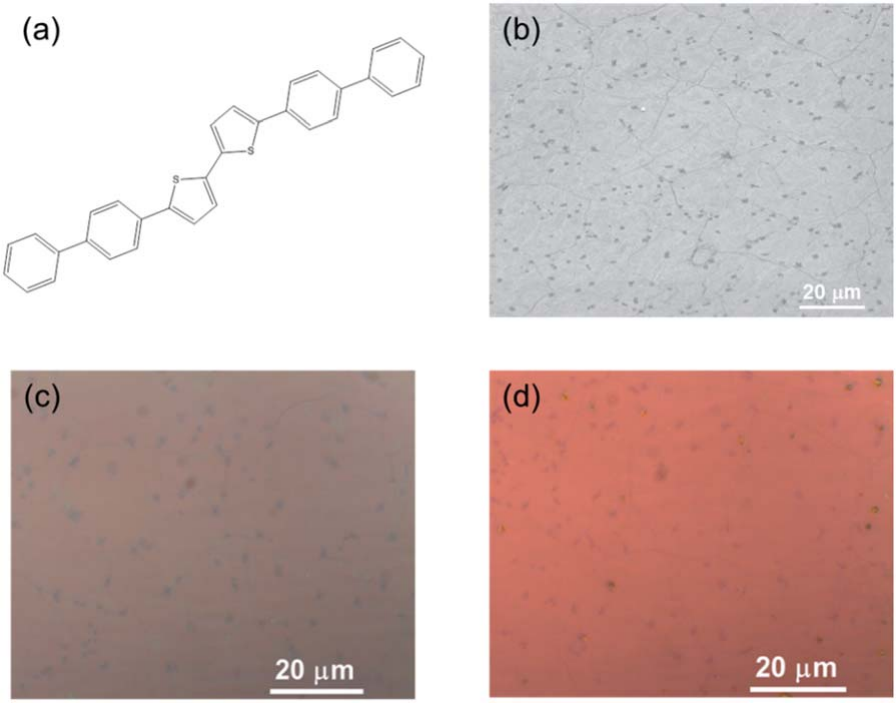
(a) Chemical structure of BP2T molecules used in the non-covalent functionalization of graphene. (b) Scanning electron microscopy image of the CVD graphene, where the dark dots denote the bilayer graphene islands. Light optical microscopy image of (c) pristine graphene and (d) graphene after non-covalent functionalization.

**Fig. 2:**
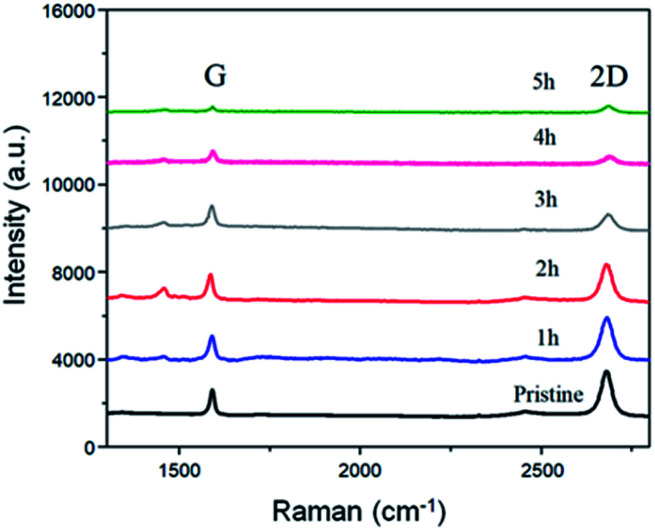
Raman comparison of the BP2T non-covalently functionalized graphene with immerging time varying from 1 h to 5 h.

Fig. 1(a) shows the chemical structure of the BP2T molecule, in which the bithiophene group lies in the center of the molecule. Here, chemical vapor deposited (CVD) monolayer graphene is utilized as the starting material and the scanning electron microscopy image is shown in Fig. 1(b). The BP2T functionalized graphene is prepared by immersing graphene into the BP2T/toluene solution, and the core idea of this non-covalent functionalization is to achieve a strong binding between graphene and BP2T molecules through π–π stacking interactions of the aromatic rings. From a comparison of the optical images (Fig. 2(c) and (d)), it is found that before and after the immersing, there is a clear color change of graphene, and such microscopic modifications on the graphene surface can be attributed to the attachment of the BP2T molecules, which leads to the variations in the light reflection.

To better understand the structure evolution of the surface after modifications, graphene with various non-covalent BP2T functionalization time ranging from 1 h to 5 h were prepared and investigated. Fig. 2 shows the Raman spectra evolution of graphene samples as a function of time. It can be seen that the starting pristine graphene shows a monolayer feature that is evidenced from the intensity ratio of 2D peak (at 2680 cm^−1^) and G peak (at 1580 cm^−1^) (*I*_2D_/*I*_G_) of more than 3, while the negligible D peak (at 1350 cm^−1^) indicates the good quality of the graphene with a very low level of defects. With the increase of the non-covalent functionalization time, both *I*_2D_ and *I*_G_ are decreased. More specifically, in the samples of immersing time of 1 h and 2 h, graphene still maintains a monolayer feature of graphene where the intensity ratio of *I*_2D_/*I*_G_ is more than 3. Nevertheless, when the immersing time is longer than 2 h, the intensity ratio of *I*_2D_/*I*_G_ tends to be 1, implying the tendency of the structure evolution towards forming a bilayer graphene.^26–29^ As for the D peak, it can be seen that all of the samples after non-covalent functionalization maintains a low intensity D peak, indicating a low defect level of the graphene, which is quite different comparing to covalent graphene functionalizations.^5,30,31^

**Fig. 3:**
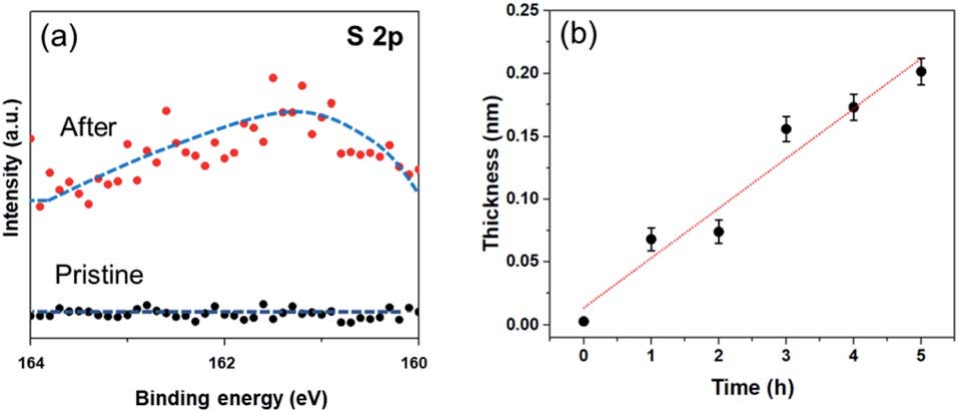
(a) XPS S 2p comparison of pristine graphene and non-covalently BP2T functionalized graphene (after 5 h) with fitting curves. (b) Evolution of the BP2T molecular thickness as a function of immersing time.

In order to investigate the formation of BP2T molecule layer on graphene surface through non-covalent π–π interactions, X-ray photoelectron spectroscopy (XPS) measurements were performed as shown in Fig. 3(a). The XPS S 2p peak comparison between the pristine and non-covalently BP2T functionalized graphene by XPS spectroscopy confirms the successful attachment of the BP2T molecules. More specifically, in pristine graphene, there is no S peak observed in the spectrum, while a prominent S peak appears after the functionalization, and this can be well attributed to the attachment of BP2T molecules containing S atoms. The film thickness measurement based on XPS was also carried out on the graphene samples with various immersing time. Because in the XPS measurements, the C signals originate from both the BP2T and graphene layer, while the O signals only comes from the SiO_2_ substrate, it is realistic to measure the film thickness of graphene *T* (*T* = *T*_graphene_ + *T*_BP2T layer_). According to the work of Cumpson and co-workers,^32,33^ the graphene thickness *T* on top of SiO_2_ substrate can be expressed as:1
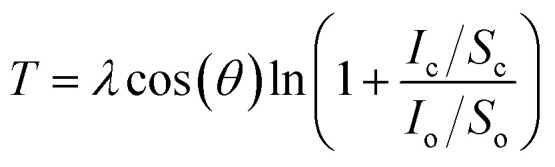
where *λ* is the mean free path of the photoelectrons, *θ* is the emission angle, *I*_c_ and *I*_o_ are the measured peak intensities of the C 1s peak of the film and O 1s peak of the substrate, respectively, and *S*_c_ and *S*_o_ are the XPS elemental sensitivity factors of carbon and oxygen. The obtained BP2T molecular thickness *T*_BP2T layer_ as a function of immersing time is shown in Fig. 3(b). It can be seen from the figure that, with the increase in the immersing time, the molecular thickness rises linearly, indicating a gradual growth of the BP2T molecule layer on top of graphene surface.

**Fig. 4:**
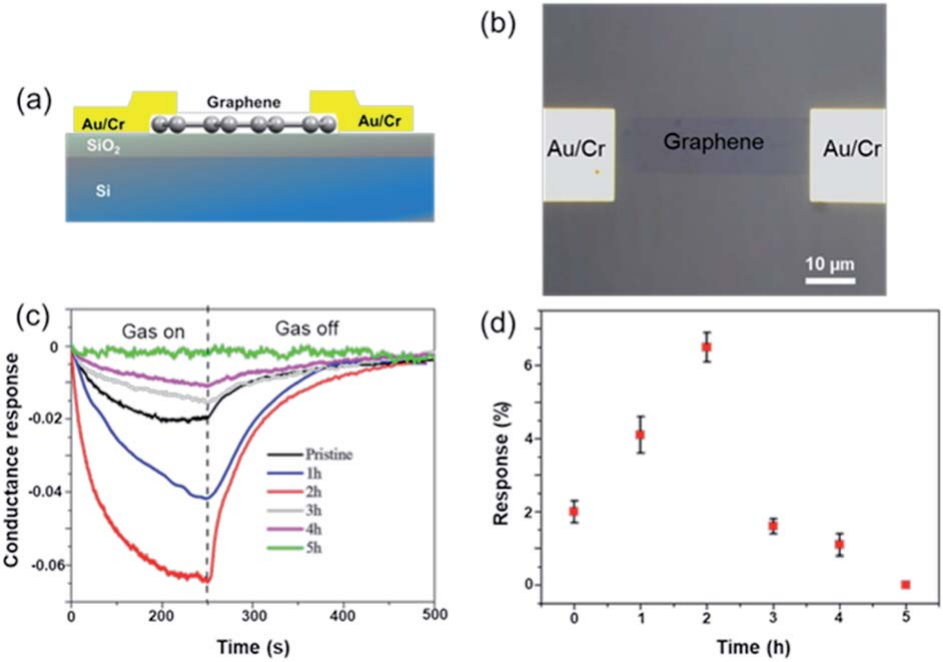
(a) Schematic illustration of the graphene FET device. (b) Light optical image of the graphene gas sensor device. The central graphene channel (40 μm long and 10 μm wide) is electrically contacted by Au/Cr pads. (c) Comparison of gas sensing experiments on graphene sensors with different non-covalent BP2T functionalization time. (d) Summarized conductance response (%) with standard derivations as error bars as a function of non-covalent BP2T functionalization time.

To facilitate the electrical analysis and gas sensing experiments, a pristine monolayer graphene is fabricated into a gas sensor and graphene is electrically contacted by Au (60 nm)/Cr (5 nm) as illustrated in Fig. 4(a). The light optical image of the fabricated graphene device with electrical contacts is shown in Fig. 4(b), and the key part of the senor is the central graphene channel with the dimension of 40 μm × 10 μm. The NH_3_ gas sensing measurements were performed in a gas sensing probe station under ambient pressure, and the current–time (*I*–*t*) characteristics of the gas sensors are recorded. To ensure reliable sensing results, the gas sensing chamber is purged by using dry N_2_ for more than 1 h prior to introducing the target gas in all measurements. The gas flow of 100 standard cubic centimeter per minute (sccm) is used in the experiment, which is controlled by a mass flow controller. Here, the conductance response *S* of the gas sensor can be defined as the normalized conductance change response as:2
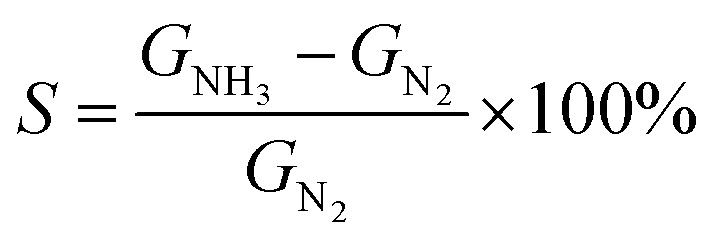
where *G*_N_2__ denotes the initial conductance of the graphene gas sensor and *G*_NH_3__ is the final conductance of the sensor under NH_3_ gas exposure. Owing to the electron donor character of the ammonia gas, the measured conductance response *S* is normally smaller than the initial conductance *G*_N_2__.^8,34^Fig. 4(c) shows a normalized conductance variation response of different graphene sensors as a function of time under the exposure of 10 ppm NH_3_ at room temperature, and Fig. 4(d) illustrates the summarized conductance response as a function of non-covalent BP2T functionalization time. From the comparison, it can be seen that, with the increase of the immersing time, the response of the graphene sensor rises considerably and the sample with 2 h immersing shows the highest sensing performance and a response of 3-fold higher than that of the pristine graphene. However, when the immersing time is longer than 2 h, the sensitivity of the graphene gas sensor declines significantly and the sensor gradually loses the sensing capabilities. Especially in the 5 h device, no significant change of *S* is observed upon the NH_3_ exposure. As for the response time, it is found from the curves that all of the devices demonstrate a similar response time of ∼250 s. Meanwhile, it is worthy to be mentioned that the recovery process of all graphene gas sensors is also very fast, around 200 s, and does not require any external treatment such as high temperature annealing or UV light illumination, showing unique sensor properties compared to conventional NH_3_ sensors that normally require higher temperature annealing for the recovery to the initial state.

Theoretical studies have predicted that graphene functionalization can result in an enhanced binding energy between graphene and target molecules, leading to a considerably increased sensitivity in gas sensing,^35,36^ and thus thiophene groups have a great potential for the detection of NH_3_ molecules.^37^ To further understand the gas sensing behavior of the non-covalently functionalized graphene, the Langmuir isotherm model is applied to estimate the binding energy between the NH_3_ molecules and functionalized graphene. In the Langmuir isotherm, the gas sensing response *S* is proportional to the gas coverage, therefore the sensitivity of the gas sensor can be written as:^38^3
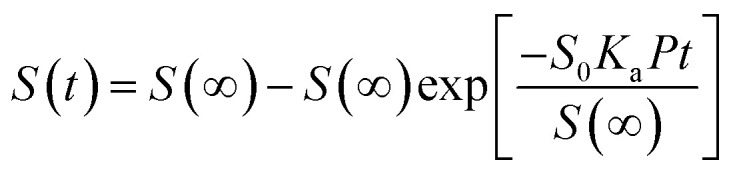
where *S*(∞) is the sensor response at equilibrium state, *S*_0_ is the saturated response, *K*_a_ is the adsorption constant, *K* is the equilibrium constant, *υ* is the attempting frequency of the gas molecules, *P* is partial pressure and *t* is response time. Under the room temperature of 300 K, the adsorption energy *E*_a_ of the gas can be expressed as:4
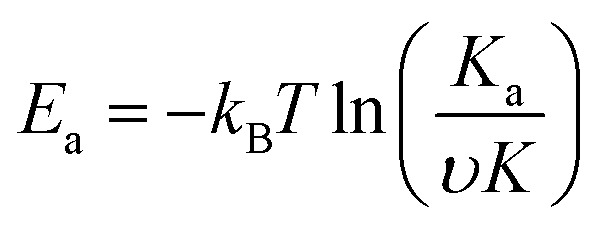
where *k*_B_ is the Boltzmann constant, *T* (300 K) is the temperature, *υ* (10^12^ s^−1^) is the attempt frequency, *K* is the equilibrium. Hereby, *S*_0_ (0.08) and *K* (0.32 Pa^−1^) can be derived by linear fitting of response *S* of as a function of partial pressure *P* according to the following formula:^10^5
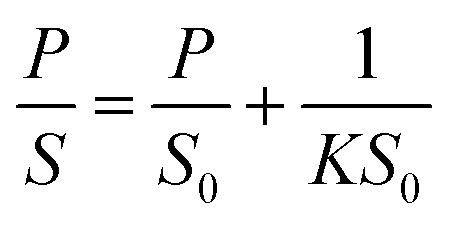


**Fig. 5:**
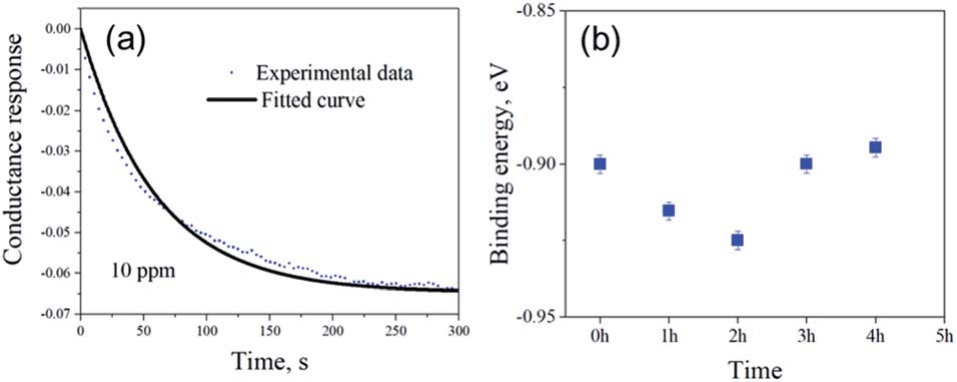
(a) Adsorption response curve of 2 h sample at ambient pressure. The continuous line shows the best fitted curve. (b) Binding energy of NH_3_ to various non-covalently functionalized graphene samples with different treatment time.

Fig. 5(a) shows the exponential fit of the adsorption curve of the 2 h sample, and the corresponding *K*_a_ can be precisely derived. Based on the equations above, it is possible to extract the binding energies of ammonia to graphene samples with different treatment time, by fitting the obtained electrical gas response curves for various samples, and the obtained binding energy as a function of immersion time is shown in Fig. 5(b). It can be seen from the comparison that the binding energy has a close relation with the gas response of the graphene. The samples functionalized for 1 h and 2 h have the strongest binding energy between the graphene and NH_3_, while sample treated with 3 h and 4 h have a lower binding energy comparing to pristine graphene and correspond well with the decreased sensitivity observed in the electrical gas sensing experiments. As for the sample after 5 h treatment, no data is derived because due to the complete loss of sensitivity.

**Fig. 6:**
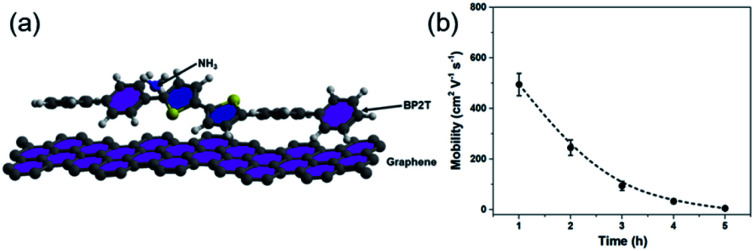
(a) DFT calculation model that is used for the calculation of the absorption. (b) Plot of the measured charrier mobility as a function of immersion time.

To further exploit the sensing mechanism of the non-covalent BP2T functionalized graphene to NH_3_ molecules, DFT calculations have been performed in this work (Fig. 6(a)). It is found from our calculation that the absorption energy of the NH_3_ molecule and bare graphene surface is 0.11 eV, which is quite low. After treatment, the absorption energy of the NH_3_ molecule and the non-covalent BP2T functionalized graphene has a significant increase to 0.45 eV. Theoretical studies have predicted that the enhanced sensitivity of graphene gas sensing is mainly attributed to the increased absorption energy of graphene after functionalization with target gas molecules. Therefore, from our calculations, the significant increased absorption energy explains well our experimental observations of the superior NH_3_ gas sensing performance of graphene. Bases on the absorption energy result from DFT, in principle, more attachments of BP2T molecules on graphene can result in more enhancements in the gas sensing responses. But it seems contradictory with our experimental data that an intermediate immersion time of 2 h shows the maximum electrical response.

To better understand this contradiction, we have carried out more electrical characterizations to understand the influence of BP2T attachment on the electrical properties of graphene and the obtained plot of carrier mobility as a function of immersion time is shown in the Fig. 6(b). It can be seen that, with the increase in the immersion time, the carrier mobility of graphene gradually decreases, which is mainly attributed to the electron scattering effect of attached BP2T molecules. This result indicates that, the physisorption of BP2T molecules gradually deteriorate the electrical performance of graphene, which means more electrical disturbances/noises will be introduced into the sensing systems, leading to the decreased electrical response in the gas sensing experiment. As for the sample with immersion time of 5 h, the carrier mobility is close to zero, indicating that the graphene has nearly lost its conductivity due to the strong electron scattering from BP2T molecules, and it agrees well with our experimental observation that the sample with immersion time of 5 h shows no nearly electrical response in the gas sensing experiments. Therefore, only from the perspective of introducing disturbances/noises, it can be concluded that, with the increase in the immersion time, the more attachments of BP2T molecules on graphene can result in the deterioration in the gas sensing responses.

Based on the discussion above, it can be deduced that when the intermediate immersion is short, the enhanced absorption energy between the ammonia and BP2T molecules plays the key role, leading to an increased gas response, while the deterioration effect is not so obvious. But when the immersion time is long, the deterioration of the electrical performance due to the attachments of BP2T molecules on graphene plays the dominating effect, resulting in a lowered gas response. So a combination of these two factors leads to an intermediate immersion time of 2 h having the highest electrical response in the gas sensing. Moreover, the binding energy is extracted from the measured electrical response of the gas sensing experiments by employing the Langmuir isotherm model, so it is a reflection of the electrical response and also a combination of the influence of the absorption energy and electrical performance due to the BP2T attachment on graphene.

## Conclusions

Herein, we have reported an approach to obtain functionalized graphene by employing non covalent stacking interactions. BP2T molecules are employed for the non-covalent functionalization and the obtained graphene shows superior ammonia sensing capacities with the sensitivity 3 times higher comparing to that of the pristine graphene, and such gas sensing result corresponds well with our derived binding energies through Langmuir isotherm model. Therefore, our work provides direct evidence of the interactions between the gas species and graphene functional groups, and the non-covalent approach can also be employed in a variety of gas detection applications.

## Materials and methods

### Materials and fabrication

Large-scale monolayer graphene (Graphenea) was synthesized by using chemical vapor deposited (CVD) approach on a copper foil^39^ at a temperatures of 1000 °C with a mixture of methane and hydrogen. The obtained graphene then will be mechanically transferred on SiO_2_ (300 nm)/Si wafer by employing a polymer-assisted method.^40^ The graphene was patterned and electrically contacted (evaporated 5 nm Cr as an adhesion layer along with a 60 nm Au) by using standard electron beam lithography (EBL) in a Nanobeam nB Series EBL system, oxygen plasma etching in an Advanced Vacuum Vision 320 Reactive Ion Etching (RIE), and physical vapor deposition (PVD) in a Lesker PVD 75 system. To prepare the molecule solution for the non-covalent functionalization, 10 mg BP2T molecules (Sigma-Aldrich) were dissolved in 10 ml of toluene. The non-covalently BP2T functionalized graphene sensors for NH_3_ detection were prepared by immersing the monolayer CVD graphene devices in the BP2T/toluene solutions for 1 h, 2 h, 3 h, 4 h and 5 h, respectively, then being washed with the toluene and followed by a blow drying.

### Characterizations

Scanning electron microscopy (SEM) and light optical microscopy (LOM) images were recorded in a Zeiss 1550 SEM and an Olympus AX70 research microscope, respectively. Raman spectroscopy was carried out in a Renishaw inVia Raman spectroscopy with a 532 nm excitation laser. X-ray photoelectron spectroscopy (XPS) was performed in a PHI Quantum 2000 spectroscopy using monochrome Al Kα radiation (1486.7 eV) with a 45° angle of electron emission. The electrical characterizations of the graphene sensor devices were carried out in an Agilent B1500 semiconductor parameter analyzer with tungsten probes, and the noise level of the current measurement was lower than 50 aA. The gas sensing experiment was performed in a home-built gas sensing probe station equipped with a gas sensing chamber and a Keithley 6430 sub-femto amp source meter inside a Faraday cage, and the whole assembly is inside fumehood. The chamber is also coupled to several mass flow controllers (Brooks 5878), which are used to regulate the speed of different gases flowing into the chamber. The electrical response of the graphene sensors to the gas can be obtained by recording the current variations as a function of gas exposure time through the source meter under the exposure of 10 ppm NH_3_ at room temperature.

### DFT calculations

To understand the binding mechanism for molecular sensing, calculations were carried out from first principles with a method based on DFT as implemented in the Siesta package.^41^ In the initial setup the BP2T molecule had perfectly aligned hexagons over graphene sheet with unit cell taken in (8,5) direction along molecule and (3,3) or 7.3830 Ang across. NH_3_ molecule was placed coordinated to sulphur atom, with preferable position to be hydrogen bonded *via* N–H⋯S and C–H⋯N bonds. All atoms were allowed to fully relax. Core electrons are modeled using Troullier–Martins^42^ soft norm-conserving pseudopotentials, with the mesh cut off was 200 Ry and Brillouin zone integration for the supercell was sampled by 1 *k*-point. The valence electrons are expanded in a basis set of local orbitals using a double-ζ plus polarization orbital (DZP) set. The GGA was used for the exchange–correlation functional.^43^

## Author contributions

Hu Li: conceptualization, formal analysis, funding acquisition, investigation, writing – original draft. Tianbo Duan: formal analysis, methodology, writing – review & editing. Omer Sher: formal analysis. Yuanyuan Han: formal analysis, methodology. Raffaello Papadakis: investigation, writing – review & editing. Anton Grigoriev: formal analysis, investigation, writing – review & editing. Rajeev Ahuja: supervision, writing – review & editing. Klaus Leifer: conceptualization, funding acquisition, methodology, writing – review & editing.

## Conflicts of interest

There are no conflicts to declare.

## Supplementary Material

RA-011-D1RA06879B-s001
