# Supplementary material for: Fabrication of BP2T functionalized graphene via non-covalent π–π stacking interactions for enhanced ammonia detection
Source: RSC Adv. 2021 Nov 8;11(57):35982–7. doi: 10.1039/d1ra06879b (PMC9043234; doi:10.1039/d1ra06879b)
Supplement: RA-011-D1RA06879B-s001 [file RA-011-D1RA06879B-s001.pdf]

This journal is © The Royal Society of Chemistry 2021

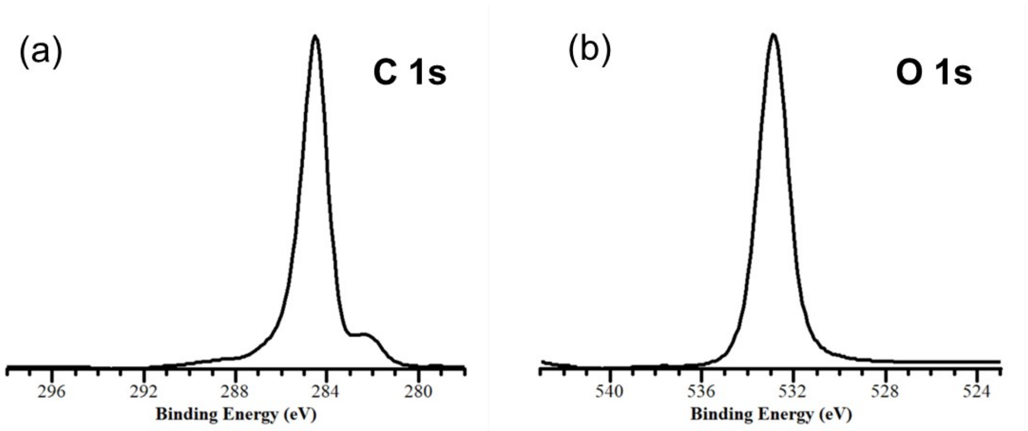

Figure S1. XPS (a) C 1s and (b) O 1s spectrum of the graphene after non-covalent BP2T functionalization.

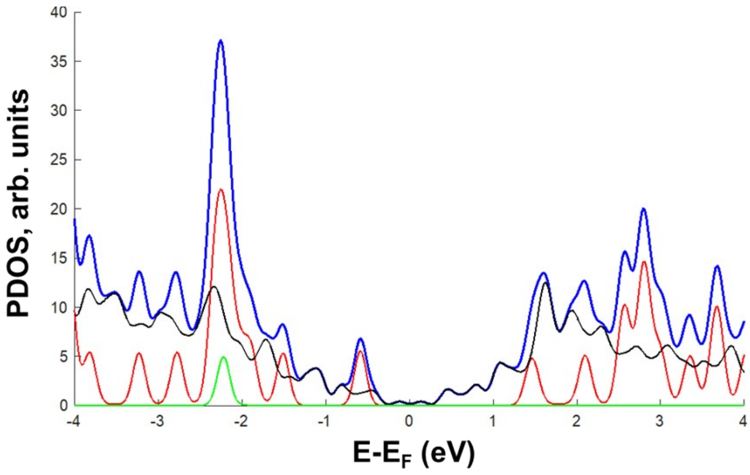

Figure S2. Projected density of states (blue) expanded into graphene (black), BP2T (red) and NH_3 contributions.

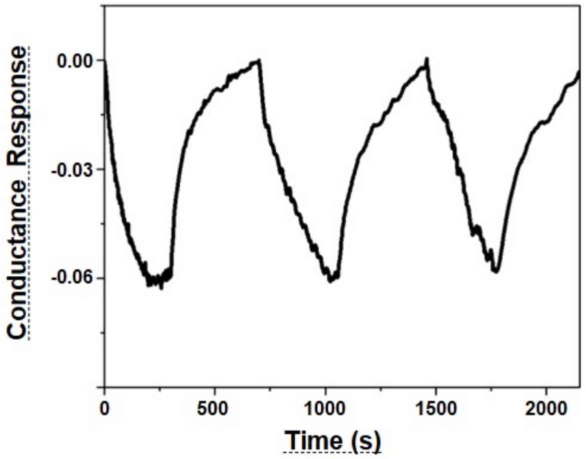

Figure **S3**. The multiple gas sensing experiment of graphene sensor with 2h non-covalent BP2T functionalization time after 3-month fabrication.

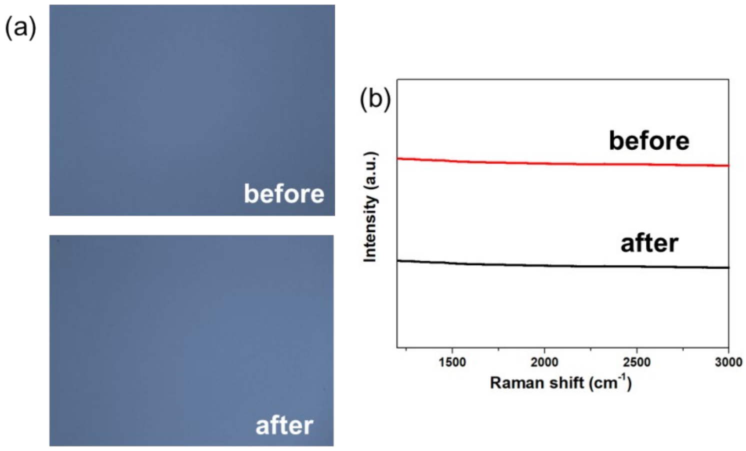

Figure **S4**. Control experiment by using a SiO_2/Si substrate immersed in the BP2T/toluene solution

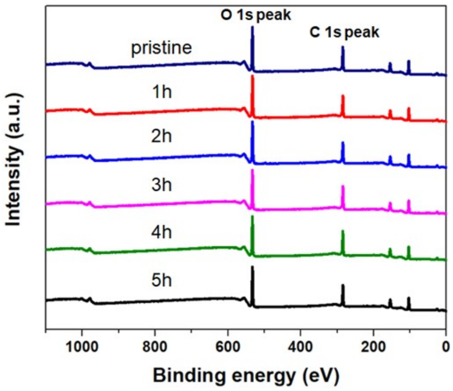

Figure **S5**. XPS survey comparison of graphene with various non-covalent BP2T functionalization time from pristine (0h) to 5h.
